# Maternal antioxidant supplementation enhances oxidative balance, milk bioactivity, and neonatal performance in Beetal goats during the transition period

**DOI:** 10.14202/vetworld.2026.111-124

**Published:** 2026-01-14

**Authors:** Gul Zaib, Kong Zhiwei, Anjaleena Yaseen, Amjad Hameed, Shakeel Ahmed Tunio, Muhammad Ismail Chughtai, Tarique Hussain

**Affiliations:** 1 College of Animal Science and Technology, Guangxi University, Nanning, China; 2 Institute of Epigenetics & Epigenomics, Yangzhou University, Yangzhou, Jiangsu 225009, China; 3 College of Veterinary Medicine, Yangzhou University, Yangzhou, Jiangsu 225009, China; 4 Animal Sciences Division, Nuclear Institute for Agriculture and Biology College, Pakistan Institute of Engineering and Applied Sciences (NIAB-C, PIEAS), Faisalabad 38000, Pakistan; 5 Nuclear Institute for Agriculture and Biology College, Pakistan Institute of Engineering and Applied Sciences (NIAB-C, PIEAS), Faisalabad 38000, Pakistan; 6 Department of Livestock and Management, Faculty of Animal Husbandry and Veterinary Sciences, Sindh Agriculture University, TandoJam, Sindh, Pakistan

**Keywords:** antioxidant supplementation, transition period, oxidative stress, enzymatic antioxidants, colostrum quality, milk biochemical profile, neonatal growth, Beetal goats

## Abstract

**Background and Aim:**

Pregnancy and early lactation in small ruminants are characterized by heightened metabolic activity and increased production of reactive oxygen species, predisposing animals to oxidative stress and reduced productivity. Despite extensive research in dairy cattle, evidence is limited for subtropical small-ruminant systems. This study evaluated whether dietary antioxidant supplementation during mid- and late-gestation improves oxidative status, milk quality, and neonatal growth in crossbred Beetal goats.

**Materials and Methods:**

Forty healthy multiparous Beetal goats were allocated to mid- (n = 20) and late-gestation groups (n = 20), each further divided into control and antioxidant-supplemented subgroups (120 mg/kg BW/day of a tocopherol–rosemary extract blend). The 90-day trial included serial blood sampling (gestation days 60–165) and milk/colostrum collection (0, 15, and 30 days postpartum). Enzymatic (Catalase [CAT], Superoxide dismutase [SOD], and Glutathione peroxidase [GPx]) and non-enzymatic antioxidants (phenolics, flavonoids, lycopene, carotenoids), total protein, Total Antioxidant Capacity (TAC), total oxidant status (TOS), and malondialdehyde (MDA) were quantified. Kid birth weight, growth, litter size, and survival were recorded. Data were analyzed using repeated-measures analysis of variance.

**Results:**

Antioxidant supplementation significantly increased CAT, SOD, and GPx activities during both gestational phases (p < 0.05), with parallel improvements in colostrum and milk enzymatic antioxidant profiles. Non-enzymatic antioxidant concentrations, including phenolics, flavonoids, lycopene, and carotenoids, were markedly elevated in treated animals across all sampling points (p < 0.05). Maternal TAC and total protein increased, while TOS and MDA were significantly reduced (p < 0.05), demonstrating enhanced redox homeostasis. Milk from supplemented goats exhibited higher antioxidant capacity and lower oxidative damage markers. Neonatal outcomes showed increased birth weight in male kids (p < 0.05), although litter size, growth rates, and survival remained unchanged (p > 0.05).

**Conclusion:**

Maternal antioxidant supplementation effectively strengthened oxidative defense mechanisms, improved colostrum and milk biochemical quality, and enhanced neonatal birth weight in Beetal goats. These findings support the strategic use of natural antioxidant blends as a nutritional intervention to mitigate periparturient oxidative stress and improve productivity under subtropical management conditions.

## INTRODUCTION

The transition period is a physiologically demanding phase marked by interconnected metabolic, endocrine, inflammatory, and immune adjustments [1]. In ruminants, this period encompasses pregnancy, parturition, uterine involution, and the onset of lactation, during which substantial physiological shifts occur [2]. These changes frequently disrupt oxidant–antioxidant homeostasis. As metabolic activity increases, oxygen demand rises [3], leading to excessive production of reactive oxygen species (ROS) and reactive nitrogen species. Overaccumulation of these reactive molecules impairs cellular metabolism and induces lipid damage [4]. Lipid peroxidation generates malondialdehyde (MDA), a widely recognized biomarker of oxidative stress [5]. In cattle and sheep, oxidative stress compromises fertility, milk yield, and immune function, increasing disease susceptibility and decreasing overall productivity, ultimately contributing to reduced reproductive efficiency, poorer lactational performance, and elevated veterinary costs [6]. Nevertheless, oxidative stress can be effectively mitigated through exogenous antioxidant supplementation that enhances both enzymatic (Catalase [CAT], Superoxide dismutase [SOD], and Glutathione peroxidase [GPx]) and non-enzymatic (vitamin E, selenium) defense mechanisms, thereby supporting normal cellular physiology [5].

Antioxidant supplementation has gained prominence for its ability to counter oxidative stress and improve progeny performance in dairy ruminants. Such supplementation enhances milk quality by lowering the somatic cell count and enriching its nutritional composition. Diets fortified with vitamins (E, A, C), minerals (selenium, zinc, manganese), and phenolic compounds (e.g., flavonoids) increase milk fat and protein content while enhancing its antioxidant potential, thereby improving nutritional value for consumers [6, 7]. Polyphenol supplementation during gestation supports maternal health by improving fetal development and reducing the likelihood of pregnancy-associated complications [8]. Polyphenol-rich diets also promote immune function, gut health, and nutrient absorption, which are critical for the growth of young ruminants during environmentally sensitive early life stages [9]. Plant-derived antioxidants such as Moringa oleifera, grape seed extract, and curcumin nanocomposites have been shown to strengthen placental function, enhance maternal antioxidant capacity, reduce oxidative stress, and improve reproductive outcomes by modulating redox and inflammatory pathways [10].

In goats, phenolic and flavonoid supplementation enhances oxidative balance, improving both milk quality and fetal development [11]. These compounds activate the Nuclear Factor Erythroid 2-Related Factor 2 (Nrf2) –Keap1 pathway, stimulating the expression of CAT, SOD, and GPx, which protect mammary epithelial cells from oxidative injury and stabilize milk composition [12]. Concurrently, flavonoids modulate PI3K/Akt and Mitogen-activated protein kinase pathways and suppress Nuclear factor kappa-B –mediated inflammation, improving uteroplacental blood flow and nutrient delivery [13]. Enhanced eNOS activity and upregulation of growth-related genes such as Insulin-like growth factor-1 and vascular endothelial growth factor further support placental efficiency, fetal growth, and elevated milk antioxidant capacity [14]. Despite these advances, research on antioxidant supplementation in small ruminants under subtropical management conditions, especially regarding maternal oxidative profiles and milk bioactivity, remains limited [15].

Despite increasing recognition of oxidative stress as a major determinant of reproductive efficiency, metabolic resilience, and neonatal viability in ruminants, current knowledge remains disproportionately centered on dairy cattle, with minimal attention to small ruminants reared under subtropical production systems. Existing studies have largely examined antioxidant supplementation during isolated physiological stages, most commonly mid-gestation or lactation, thereby overlooking dynamic changes in oxidative status across the entire transition period. Moreover, few investigations have simultaneously evaluated enzymatic and non-enzymatic antioxidant responses, along with milk biochemical attributes and neonatal outcomes, which are critical indicators of maternal–offspring health. Beetal goats, a key dual-purpose breed widely raised in South Asia, face heightened metabolic challenges due to high ambient temperatures, forage variability, and increased oxidative load during late-gestation and early lactation. However, the oxidative physiology of this breed and its responsiveness to dietary antioxidant interventions during periparturient stress remain poorly documented. In particular, no studies have comprehensively assessed how natural antioxidant blends influence maternal redox homeostasis, milk antioxidant capacity, and kid growth when administered across both mid- and late pregnancy phases.

This study aimed to evaluate the effects of maternal antioxidant supplementation during the transition period on oxidative balance, milk biochemical quality, and neonatal performance in crossbred Beetal goats. Specifically, the research sought to (i) characterize changes in enzymatic (CAT, SOD, and GPx) and non-enzymatic (phenolics, flavonoids, lycopene, carotenoids) antioxidant markers across mid- and late-gestation; (ii) assess how dietary antioxidants influence colostrum and milk antioxidant potential and oxidative stability; and (iii) determine whether improved maternal redox status translates into measurable benefits in kid birth weight, growth parameters, and survival. By integrating longitudinal biochemical profiling with productive performance indicators, the study intended to generate a holistic understanding of how natural antioxidant supplementation could serve as a practical nutritional strategy to mitigate periparturient oxidative stress and enhance the productivity of goats managed under subtropical conditions.

## MATERIALS AND METHODS

### Ethical approval

The study protocol was reviewed and approved by the Institutional Animal Care and Use Committee at Nuclear Institute for Agriculture and Biology (NIAB), Pakistan (Approval No. NIAB/ASD/11-2025). (Approval No. SAU-FAHVS-00125). All procedures complied with international guidelines for the ethical care and use of animals in research.

### Study location and animal management

The experiment was conducted from December 2019 to February 2020 at Chak Jhumra experimental goat farm, Faisalabad, Pakistan. Forty healthy, multiparous crossbred Beetal goats (average body weight [BW]: 40.0 ± 0.5 kg) in third parity with a body condition score above 3.5 were selected. No previous reproductive failures were recorded. Goats were individually housed in well-ventilated pens (1.5–2.5 m² per animal) bedded with straw (10–15 cm depth), with bedding replaced every 2–3 days.

Environmental conditions, including temperature (10°C–24°C), relative humidity (50%–70%), and air quality (ammonia <10 ppm; CO_2_ <3,000 ppm), were monitored daily. A natural photoperiod of 11–13 h of light was maintained. All animals had free access to fresh water and mineral blocks, and pasture grazing was offered twice daily.

### Experimental design and animal allocation

Goats were allocated to two physiological categories: mid-pregnancy (n = 20) and late pregnancy to early lactation (n = 20). Each category was subdivided into a control group and a treatment group antioxidant supplementation (Loxidan at the rate of 120 mg/kg BW per day, Kaesler Nutrition GmbH, Germany). Randomization was based on parity and BW to ensure uniform distribution across groups.

### Diet composition and supplementation protocol

**Figure 1:**
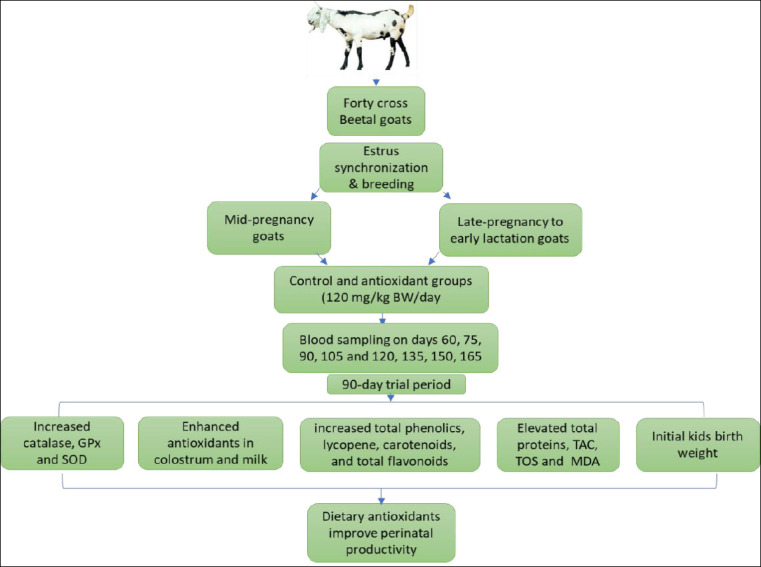
Schematic diagram of the experiment.

All animals received a basal diet formulated according to NRC (2007) [16] recommendations, consisting of mung bean straw, wheat bran, rice bran, corn, linseed, vitamins, and a mineral premix. The antioxidant supplement (Loxidan, Kaesler Nutrition GmbH) contained a mixture of tocopherols (E-306) and rosemary extract. The supplement was mixed daily with the concentrate portion and fed for 90 days at a dosage of 120 mg/kg BW. Daily feed intake and refusals were recorded. The compositions of the antioxidant-enriched and basal diets are provided in Tables 1 and 2. The Schematic diagram of the experiment is illustrated in Figure 1.

**Table 1:** Composition of the antioxidant-enriched diet.

Antioxidant items	Mean ± SEM
TPC (μM/g)	88300 ± 1418.919
TAC (μM/g)	14.654 ± 1.663
TF (Ru equivalent) (μg/g)	8268.02 ± 92.13
Ascorbic acid (μg/g)	676.333 ± 2.682
Total carotenoid content (mg/g)	1.342 ± 0.181

SEM = Standard error of the mean, TPC = Total phenolic content, TAC = Total antioxidant capacity, TF = Total flavonoids

**Table 2:** Composition and proximate analysis of experimental diets for cross-breeding Beeetal goats.

Diet ingredients (%)	Percentage
Basal diet	
Rice bran	2
Corn	5
Mung bean grains	25
Mung bean straws	19
Wheat bran	15
Wheat grains	11
Wheat straws	21
Dicalcium Phosphate	1.6
Premix of vitamins and minerals	0.4
**Proximate analysis (%)**	
Dry matter	91.7
Crude protein	13.2
Ash	3.2
Non-fiber carbohydrates	27.6
Acid detergent fiber	18.03
Neutral detergent fibers	53
Ether extract	3
Net energy (MJ/kg DM)	5.88

### Reproductive management and pregnancy confirmation

Estrus synchronization was performed with two prostaglandin injections (Cloprostinol; Synchromate®, 125 μg; Bremer, Germany) administered 12 days apart. Does were mated with fertile bucks, and pregnancy was confirmed on day 30 post-breeding using ultrasonography (ExaGO, Anshili, China) with a 7.5 MHz linear probe. Antioxidant supplementation was administered from gestational days 60–105 for mid-pregnant goats and from days 120–165 for late pregnancy to early lactation goats.

### Blood, colostrum, and milk sampling procedures

Blood samples (5 mL) were collected via jugular venipuncture at gestational days 60, 75, 90, and 105 for mid-pregnancy goats, and days 120, 135, 150, and 165 for late pregnancy to early lactation goats. Samples were centrifuged (1000 × *g*, 10 min, 4°C), and serum was stored at −20°C until analysis.

Colostrum and milk samples were collected on day 0 (colostrum), day 15, and day 30 postpartum using aseptic hand-milking. Samples were defatted by centrifugation (2,500 × *g*, 15 min) and stored at −20°C. Freeze–thaw cycles were minimized, and all samples were analyzed within three months. Sequential sampling enabled the tracking of enzymatic changes and oxidative trends throughout gestation and lactation, an approach rarely documented in small ruminants.

### Biochemical analyses

#### Total antioxidant capacity (TAC)

TAC was determined using the 2,2’-azino-bis (3-ethylbenzothiazoline-6-sulfonic acid) [ABTS], Sigma-Aldrich (St. Louis, MO, USA) decolorization assay following Re *et al*. [17]. The ABTS radical cation was prepared by reacting 7 mM ABTS with 2.45 mM potassium persulfate and incubating for 12–16 h. A diluted ABTS solution (absorbance 0.700 ± 0.020 at 734 nm) was mixed with samples, and absorbance was measured after 6 min at 30°C. Results were expressed as Trolox equivalents.

#### Enzymatic antioxidants (CAT, SOD, and GPx)

CAT activity was measured spectrophotometrically (Hitachi U-2800, Tokyo, Japan) at 405 nm using hydrogen peroxide as the substrate [18]. SOD activity was measured by the pyrogallol autoxidation method, with absorbance recorded at 420 nm every 3 s for 5 min [19]. GPx activity was determined using the guaiacol oxidation assay at 470 nm, with results expressed as nmol guaiacol oxidized per minute [20].

#### Non-enzymatic antioxidants (ascorbic acid, phenolics, flavonoids)

Ascorbic acid concentration was quantified using a modified colorimetric procedure with trichloroacetic acid (Sigma-Aldrich, MO, USA) deproteinization and the Folin–Ciocalteu reagent [21]. Total phenolics were measured using the Folin–Ciocalteu method at 760 nm and expressed as gallic acid equivalents [22]. Flavonoids were quantified using the aluminum chloride colorimetric assay at 415 nm [23].

#### Protein concentration and lipid peroxidation (MDA)

Total protein was quantified spectrophotometrically using bovine serum albumin standards and tert-butyl phenyl ether reagent [24]. MDA levels were determined by High-performance liquid chromatography after a thiobarbituric acid reaction, using a C18 reverse-phase column and fluorescence detection (λ_ex = 527 nm, λ_em = 551 nm) [25]. Results were expressed as nmol/g protein.

### Statistical analysis

Data normality was assessed using the Shapiro–Wilk test. A mixed-model analysis of variance with repeated-measures evaluated treatment, time, and treatment × time interactions, with individual goats as random effects (SPSS v.20.0; IBM Corp., NY, USA). Fisher’s least significant difference test was used for post hoc comparisons. Results are presented as mean ± Standard error of the mean, and differences were considered significant at p < 0.05. Chi-square tests analyzed categorical variables (litter size and survival rate). Figures were generated using GraphPad Prism v.9 (GraphPad software, USA).

## RESULTS

### Enzymatic antioxidant responses (CAT, SOD, and GPx)

**Figure 2:**
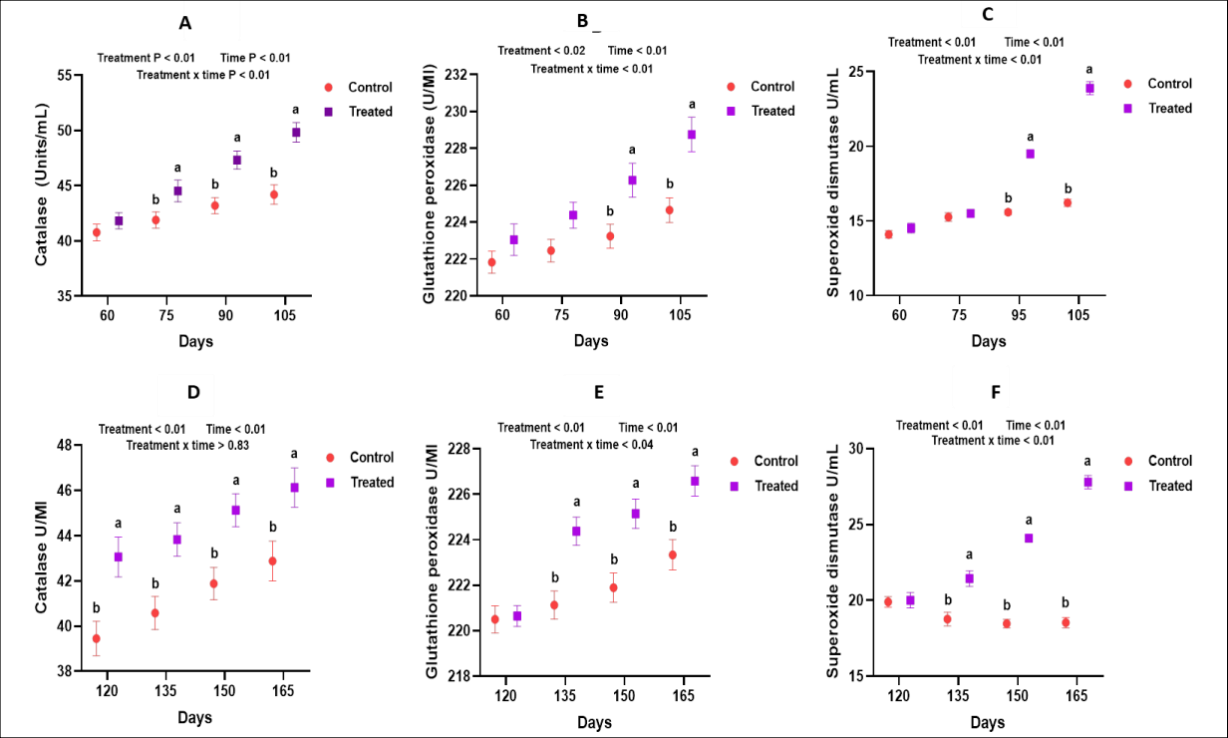
Effect of an antioxidant diet on the serum enzymatic antioxidant profile of goats during mid-pregnancy and late pregnancy.

**Figure 3:**
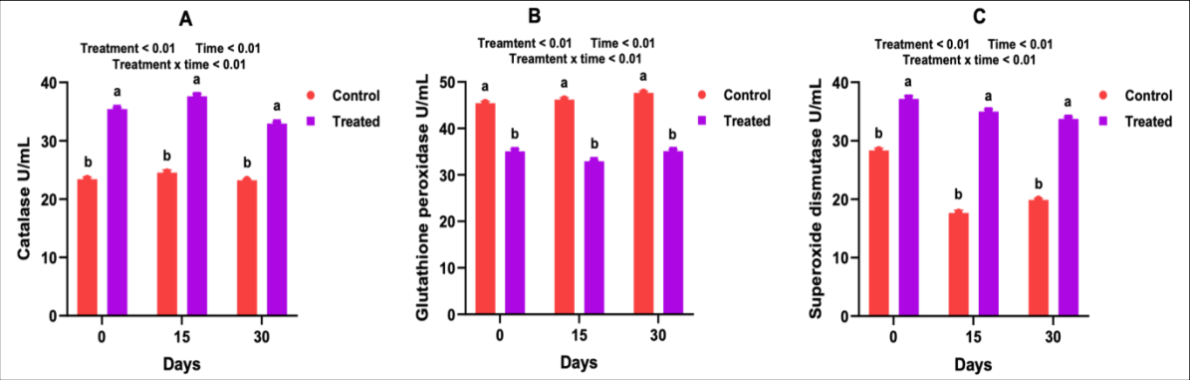
Influence of antioxidants on milk enzymatic antioxidant profile in goats.

Antioxidant supplementation markedly influenced the serum enzymatic antioxidant profile of goats. CAT activity increased significantly in the treated group on gestational days 75, 90, and 105 during mid-pregnancy (p < 0.05; Figure 2A). During late pregnancy and early lactation, CAT activity remained consistently higher than in the control group across all sampling days (p < 0.05; Figure 3A). GPx and SOD activities followed a similar pattern. The highest activities were observed on days 90 and 105 in the mid-pregnancy–treated group (Figures 2B and 2C), whereas in late pregnancy to early lactation, GPx and SOD values increased on days 135, 150, and 165 (p < 0.05; Figures 3D–3F). In colostrum and milk, CAT and SOD concentrations were significantly higher in the supplemented animals than in the controls (p < 0.05; Figure 3A and 3C). However, GPx activity in milk did not differ significantly between the two groups (p > 0.05; Figure 3B).

### Non-enzymatic antioxidant profiles (phenolics, lycopene, carotenoids, and flavonoids)

**Figure 4:**
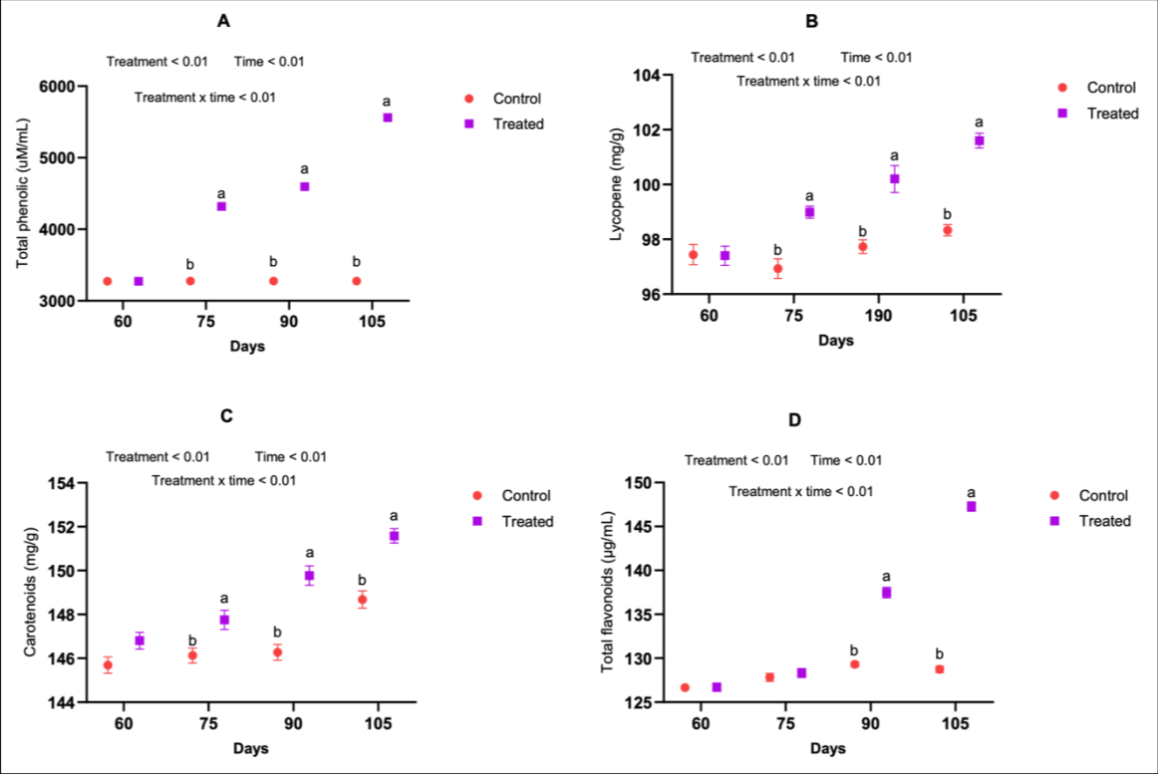
Effect of an antioxidant diet on the serum non-enzymatic antioxidant profile of goats during mid-pregnancy.

**Figure 5:**
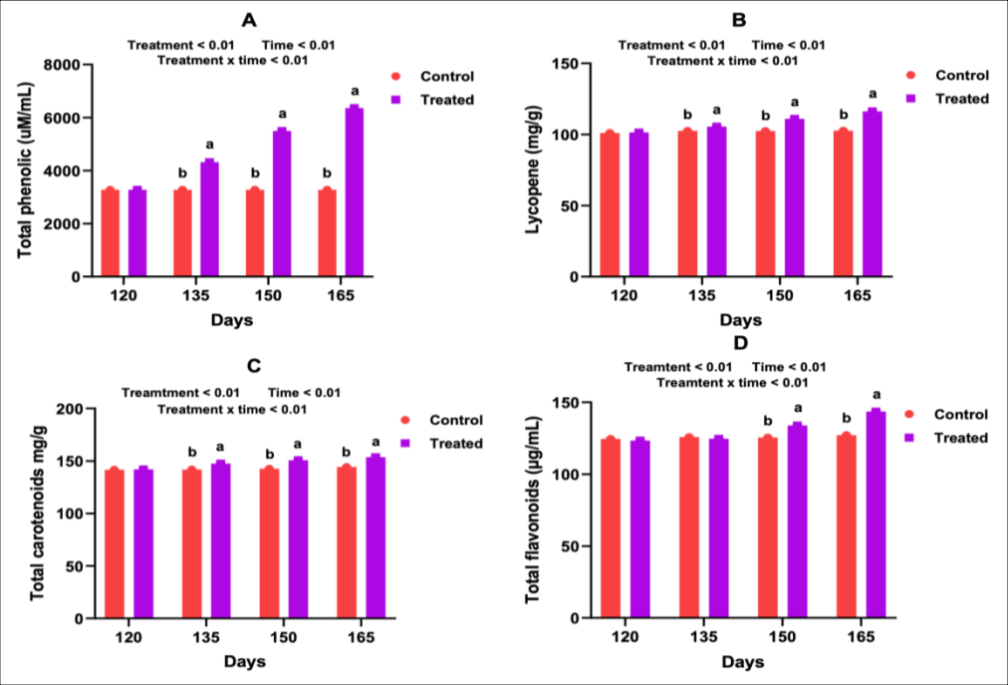
Influence of an antioxidant diet on the serum non-enzymatic antioxidant profile of goats during late pregnancy.

Supplementation resulted in significant increases in serum concentrations of total phenolics, lycopene, carotenoids, and total flavonoids. These effects were observed consistently across mid-pregnancy on days 75, 90, and 105 (Figures 4A–D) and during late pregnancy to early lactation on days 135, 150, and 165 (p < 0.05; Figures 5A–D). These findings demonstrate that antioxidant supplementation enhanced the availability of multiple non-enzymatic antioxidant compounds throughout the transition period.

### Biochemical indicators of oxidative status (protein, TAC, total oxidant status [TOS], and MDA)

**Figure 6:**
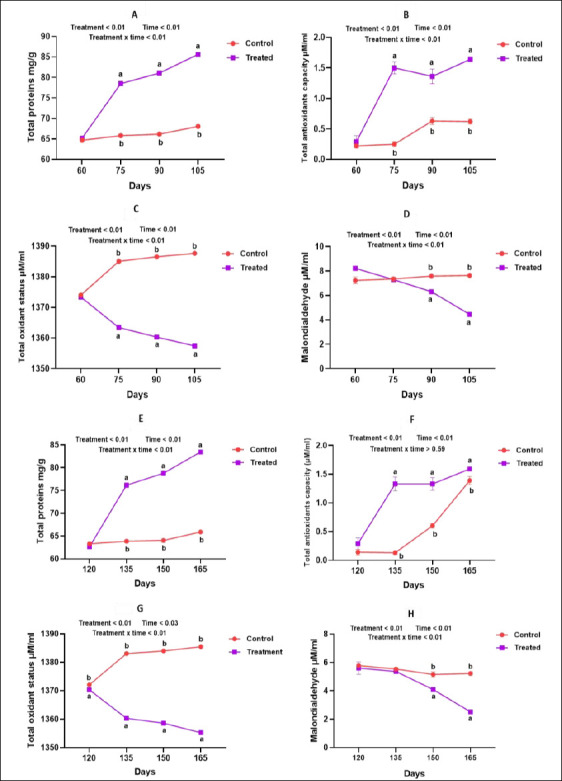
Impact of antioxidants on serum biochemical profile during mid- and late pregnancy in goats.

**Figure 7:**
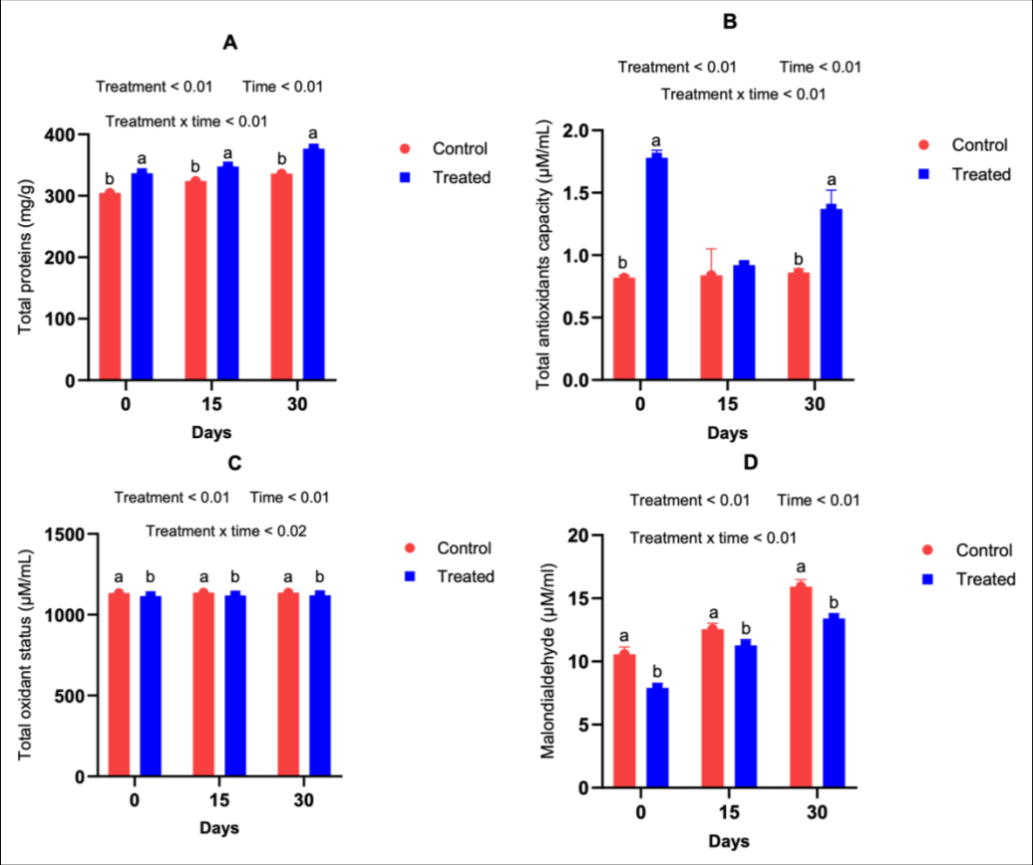
Influence of antioxidants on the biochemical profile of goat milk.

Total protein concentration and TAC were significantly higher in supplemented goats during both mid-pregnancy (days 75, 90, 105; Figures 6A and 6B) and late pregnancy to early lactation (days 135, 150, 165; Figures 6E and 6F) (p < 0.05). Conversely, TOS and MDA levels were significantly reduced in the treated groups across both physiological stages (p < 0.05; Figures 6C, 6D, 5G, and 6H), indicating improved oxidative balance. Milk biochemical parameters followed the same trend. Milk protein content and TAC were significantly higher in the supplemented groups (p < 0.05; Figures 7A and 7B), whereas milk TOS and MDA concentrations were significantly lower (p < 0.05; Figures 7C and 7D), reflecting enhanced milk oxidative stability.

### Growth performance of beetal kids

**Figure 8:**
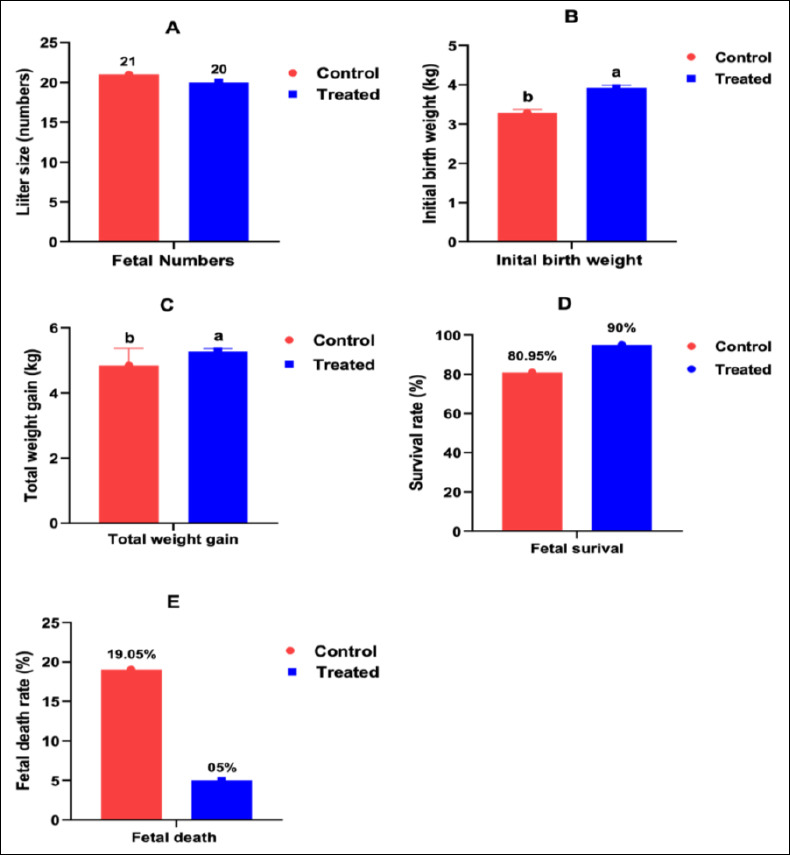
Effect of antioxidant supplementation on the growth of Beetal goat kids.

Growth performance indicators are shown in Figure 8. Antioxidant supplementation did not affect litter size (Figure 8A), initial birth weight of female kids (Figure 8C), total weight gain of male (Figure 8D) or female kids (Figure 8E), or postnatal survival (Figure 8F). However, a significant improvement was observed in the initial birth weight of male kids born to supplemented dams (p < 0.05; Figure 8B). No additional growth advantages were detected.

## DISCUSSION

### Role of antioxidant supplementation in sustainable goat production

Antioxidant supplementation supports sustainable goat production by mitigating climate-induced metabolic stress, reducing oxidative burden, and improving neonatal viability, consistent with the One Health framework for animal productivity and welfare [26]. Oxidative species generated during gestation can damage fetal tissues, underscoring the importance of prenatal nutrition for offspring developmental resilience. Factors such as oxidative stress, micronutrient deficiencies, and environmental stressors influence fetal susceptibility and health outcomes [27]. The present study demonstrates that antioxidant supplementation enhances enzymatic defenses (CAT, SOD, and GPx) and reduces MDA, thereby improving maternal redox homeostasis during the transition period [28].

### Metabolic demands of gestation and antioxidant defense

During advanced pregnancy and early lactation, animals experience heightened metabolic demands associated with fetal growth and milk synthesis, leading to increased oxygen consumption [29]. Previous research in goats shows elevated ROS biomarkers during gestation, indicating oxidative stress and the need for a robust antioxidant defense [30, 31]. Our findings confirm that prenatal antioxidant supplementation modulates oxidative stress indicators in maternal blood and milk, supporting enzymatic systems that counteract ROS. CAT, GPx, and SOD are primary enzymatic defenses, neutralizing ROS and preventing cellular damage [32]. The observed increase in CAT activity suggests enhanced free radical breakdown.

Unlike studies conducted in controlled environments, this work evaluates a commercial antioxidant blend in naturally grazing Beetal goats, integrating both biochemical and productive outcomes. Under metabolic stress, antioxidants upregulate catalytic enzyme activity [33]. GPx and SOD are particularly important in detoxifying hydrogen peroxide and preventing oxidative damage to proteins, nucleic acids, and lipids [34]. Their activity depends on glutathione availability [35]. Although initially non-significant, GPx and SOD levels later increased in supplemented goats, consistent with findings in buffaloes showing elevated antioxidant enzyme activity following dietary supplementation [36].

### Oxidative stress, lipid peroxidation, and antioxidant effects

ROS are natural by-products of metabolism; excess ROS oxidize cellular components, impairing structure and function [37]. In untreated animals, elevated TOS reflects an inability to neutralize free radicals during gestation [38]. Increased metabolic rate in late pregnancy further elevates oxidant production, contributing to disrupted cellular physiology [39]. MDA, a key indicator of lipid peroxidation, increases under oxidative stress due to hydrogen peroxide accumulation [40]. In this study, supplemented goats exhibited significantly reduced MDA levels, consistent with a previous study showing that flavonoid supplementation decreases pregnancy-induced oxidative damage [41].

Protein depletion is common during the peripartum period due to fetal amino acid demands [42]. The significantly lower total protein levels in the control group suggest greater oxidative and metabolic strain. The higher protein concentrations in supplemented goats may reflect enhanced enzyme synthesis and antioxidant status.

### Enhancement of non-enzymatic antioxidant profiles

Biological systems possess multiple protective strategies to neutralize harmful ROS [43], yet antioxidant capacity naturally declines during pregnancy [44]. Phenolic compounds and flavonoids, often deficient in grazing diets, suppress ROS formation, enhance enzymatic reactivity, and strengthen antioxidant defense pathways [45]. Vitamins and plant-derived antioxidants work synergistically to support fetal development, reproductive performance, and maternal oxidative balance [46]. The present findings confirm elevated phenolic and flavonoid levels following supplementation, demonstrating attenuation of pregnancy-induced oxidative stress.

Lycopene and carotenoids, potent plant-derived antioxidants, protect tissues from oxidative damage through complementary mechanisms [47]. Previous studies report reduced levels under pathological conditions [48]. In our study, supplementation significantly increased lycopene and carotenoid concentrations, indicating enhanced antioxidant capacity from combined phenolic and pigment sources.

### Maternal antioxidant status and milk quality

The placenta serves as the critical interface for maternal–fetal nutrient exchange, while colostrum and milk are major postnatal sources of immune and antioxidant protection [49, 50]. Although colostrum contains ROS-generating mechanisms involved in microbial defense, the macromolecules it delivers are highly susceptible to oxidative injury [51]. Maternal diet markedly influences colostrum quality, particularly protein composition and antioxidant potential, which are essential for neonatal immunity and gastrointestinal development [52]. Improved maternal oxidative balance enhances the transfer of immunoglobulins and bioactive compounds to offspring [53].

This study demonstrated that antioxidant supplementation increased milk protein levels, consistent with earlier findings linking antioxidant intake to improved milk macronutrient composition [54]. High-yielding animals often exhibit reduced milk antioxidant potential due to oxidative stress in mammary somatic cells, leading to increased MDA [55]. Dietary antioxidants reduce oxidative damage, reduce the incidence of intramammary infections, and enhance milk TAC [56]. Our results align with studies showing improved antioxidant capacity following Moringa supplementation [55].

### Antioxidant influence on neonatal oxidative protection

Newborns face significant oxidative challenges when transitioning from a hypoxic intrauterine environment to ambient oxygen conditions [56]. Adequate antioxidant support is therefore critical. Mammary-derived enzymes such as SOD and GPx contribute significantly to milk’s antioxidant defense [57, 58]. In this study, antioxidant supplementation strengthened SOD activity in colostrum and milk, consistent with reports showing that these enzymes synergistically improve milk oxidative stability [58]. GPx and CAT activities were also enhanced, supporting nutrient preservation and milk quality, in agreement with Paraskevakis’s observations [60–62].

### Effects on reproductive outcomes and neonatal growth

Supplementation increased birth weight in male offspring during late pregnancy and early lactation, supporting the role of antioxidants in reducing oxidative stress and promoting fetal growth [63]. However, litter size, survival rate, and postnatal growth were not significantly affected, likely due to genetic variability and environmental influences that are difficult to modify through dietary interventions [64].

## CONCLUSION

This study demonstrates that maternal antioxidant supplementation during the transition period significantly improves oxidative balance, enhances milk biochemical quality, and supports neonatal development in crossbred Beetal goats. Supplemented animals showed increased enzymatic antioxidant activities (CAT, SOD, and GPx), higher non-enzymatic antioxidant levels (phenolics, flavonoids, lycopene, carotenoids), elevated total protein and TAC, and markedly reduced TOS and MDA in both serum and milk. These physiological benefits translated into improved offspring outcomes, particularly higher birth weights in male kids, highlighting the strong influence of maternal redox status on neonatal performance.

The practical implications of these findings underscore the value of incorporating natural antioxidant blends into gestational diets as an accessible strategy to mitigate oxidative stress, enhance milk quality, and promote healthier progeny. This approach is especially relevant for goats managed under subtropical conditions, where climatic fluctuations and pasture-based feeding systems predispose animals to oxidative stress. The evidence presented offers producers and nutritionists a non-pharmaceutical, cost-effective means to improve herd productivity and resilience.

A notable strength of this study is the longitudinal evaluation of mid- and late pregnancy, providing a detailed understanding of oxidative dynamics during the transition period. By integrating biochemical markers from serum, colostrum, and milk with reproductive and neonatal outcomes, this work presents a comprehensive assessment rarely documented in small-ruminant research. Conducting the study in naturally grazing Beetal goats under field-relevant conditions further enhances the applicability of the findings.

However, the work is limited by its moderate sample size and the absence of molecular-level analyses that could confirm specific antioxidant-regulated pathways, such as Nrf2 activation or changes in related gene expression. Additionally, postnatal growth beyond the early life stage was not monitored, preventing conclusions about long-term developmental impacts.

Future studies should incorporate transcriptomic or metabolomic profiling to elucidate underlying mechanisms, evaluate dose–response effects of natural antioxidants, and extend the research to different breeds, environmental settings, and feeding systems. Long-term monitoring of offspring growth, immunity, and reproductive success would also deepen understanding of intergenerational benefits.

In conclusion, maternal antioxidant supplementation represents a viable nutritional strategy to enhance metabolic resilience during the transition period by improving redox balance, enriching milk bioactivity, and promoting improved neonatal health, thereby contributing to greater reproductive efficiency and productivity in goat production systems.

## DATA AVAILABILITY

The supplementary data can be obtained from the corresponding author upon request.

## AUTHORS’ CONTRIBUTIONS

GZ: Laboratory work, data extraction, and drafting of the manuscript. KZ, TH: Study design, conceptualization, administration of the project, and editing of the manuscript. AY, and AH: Data aggregation, statistical analysis, and editing of the manuscript. SAT and MIC: Data analysis and editing of the manuscript. All authors have read and approved the final version of the manuscript.

## COMPETING INTERESTS

The authors declare that they have no competing interests.

## PUBLISHER’S NOTE

Veterinary World remains neutral with regard to jurisdictional claims in the published institutional affiliations.

## ACKNOWLEGDMENTS

The authors are grateful to Guangxi Natural Science Foundation Program (2024GXNSFAA010021) for funding the study.
